# Extra Supplement—The Nursing Mirror

**Published:** 1889-09-21

**Authors:** 


### The Nursing Mirror.

All Contributions for this Supplement should be addressed to the Editor, The Hospital, 139-140, Salisbury Court, Fleet Street, London, E.C., and should have the word "Nursing" plainly written in left-hand top corner of the envelope.

### En Passant.

OUR PRACTICAL QUEEN.—One of the first nurses to receive the Royal Red Cross was Miss Weldon, and when she knelt before the Queen in her Netley uniform, the Queen touched her grey gown, and said, "I like this stuff very much, if only it would wash." "May it please your Majesty, it washes excellently," replied Miss Weldon promptly. The Queen apparently admired the prompt answer, for when she visited Netley shortly after she particularly asked to see Miss Weldon again.

EVELINA HOSPITAL.—This hospital for children is well known as a training school for nurses. Miss Alice Cross is lady superintendent, and Miss S. A. Grigg is ward sister. The work of the hospital is carried on by the committee of management and the honorary medical and surgical staff, aided by a resident medical officer, the lady superintendent, the ward sister, and the secretary, together with an adequate staff of nurses and lady pupils. Last year an appeal was made for funds to reboard some of the floors. A most gratifying feature in connection with this appeal was the collection by the nurses, past and present, of a sufficient sum to entirely cover the expense of reflooring one of the smaller wards, thus showing how greatly the nursing staff appreciated the improvement.

NURSE OR DOCTOR.—Dr. Richardson, in his small book on the "Duties and Conduct of Private Nurses," is very hard on those nurses who try and usurp the doctor's place. It is a fault which is more prevalent than intoxication amongst present nurses, but a fault which is scarcely less disastrous. The drunken Sairey at most left undone her duties, whereas the presumptuous nurse does active mischief. "The medical profession is now open to women," writes Dr. Richardson, "but I would urge upon nurses to remember that when engaged in the care of a case, their services are required as nurse, not physician. If you decide to enter the medical profession, you are then the physician and not the nurse. Do not attempt to be both." Some nurses are foolish enough to institute comparisons between one doctor and another, and while not actually usurping their place, yet taking upon themselves to criticise the doctor's treatment or manner. These faults are the subject of frequent complaints, and therefore we desire to keep them before our readers as a warning.

MIDWIFERY IN AMERICA.—The history of midwifery training schools in the States is highly interesting. In 1832 a lying-in charity was established at Philadelphia, and six years later this was merged into a nurse school with eight pupils. In 1863 provision was made for a separate home for the pupil nurses, and still later a small hospital was established. There are about a dozen nurses now connected with this charity, and some hundreds of applications for their services are received annually. The Missouri School of Midwifery was established at St. Louis in 1875, and trains nearly twenty pupils yearly. At Brooklyn there is a school in connection with the Homœopathic Maternity Hospital. The course lasts one year, and includes instruction in the care of infants. New York boasts a college of midwifery where instruction is given in all civilised languages. Three months of lectures and demonstrations are followed by three months of practical work, and then, if a stiff examination is well passed, a diploma is granted. The fee for the course is one hundred dollars.

STRATFORD-ON-AVON. — At the late meeting of Stratford-on-Avon Hospital authorities, Mr. Mason, the medical officer, read part of a letter from Miss Lydia Smith, who, our readers may remember, left Stratford some months since to take up missionary work in Africa. Miss Smith wrote: "If you knew how much we are wanted out here, you would not regret sparing us (that is to say, nurses with a knowledge of surgical work)." Miss Smith's successor, Miss Sackett, has been hard at work entirely reorganising the nursing staff at Stratford-on-Avon; and several well-known faces are now missing at the hospital. The governors highly approve of Miss Sackett's endeavours.

OVERCROWDING.—The evil results of bad ventilation on maternity cases are well known, but are brought to the fore again by a note written by Dr. Clayton. In a midwifery department 15 patients were admitted during a week, 19 were discharged, one had died, and 26 remained in the department. Thirteen children had been born in the institution during the week, none of which had died. Dr. W. M. Clayton, medical officer of the midwifery department, submitted the following report: "The general health of the midwifery department this week, is unfortunately most unsatisfactory. Owing to extreme pressure on the accommodation of the hospital for some weeks, there has been no opportunity of properly airing and ventilating the wards, and, as a consequence, there has been a run of high temperatures for the past eight or ten days, especially in wards No. 5 and No. 6. Some days ago, two patients, named Mary O'Neil and Annie Procter, developed suspicious symptoms, and were immediately isolated. The symptoms becoming more pronounced, it was considered advisable to remove them to the cottage, so that isolation could be more thoroughly carried out, and special nurses were told off to attend to them exclusively. Both patients had a perfectly normal confinement, so there is no way to account for these symptoms except through overcrowding and want of ventilation in the wards."

HEROISM.—In *Scribner's Magazine* is an account of a Russian woman, Madame Anna Pavlovara Korba, who was a nurse, but was also something more. In the small town of Minsk she organised a society and raised a fund for promoting popular education and aiding poor students. She entered the hospitals as a sister of mercy among the wounded, and went through the Russo-Turkish campaign as a Red Cross nurse. She became an enthusiastic admirer and advocate of the wretched Russian peasant, and set herself to promote a better state of things. Opposed at every turn by officials, she joined the party of "The Will of the People." She was at length arrested, and asked on her trial if she had any last words to say in her defence, she made a remarkable speech, in which she claimed that the "Will of the People Party" was not one of terror. If it adopted terrorism, it was because that was the only possible method of attaining the objects set before it. According to the fundamental laws of the Empire, no one has a right to advocate any change in the existing Imperial form of government, or even to think of such a thing. "We do not adhere obstinately to terrorism," concluded Madame Korba; "the hand that is raised to strike will instantly fall if the Government will change the political conditions of life." The sentence on the noble woman was twenty years of penal servitude. It is impossible for us in our free England to comprehend the horrors that may surround a woman's life in Russia.

### Physiology for Nurses.

### NO. X.—THE MECHANISM OF MOVEMENT.

We must now speak of the "mechanism of movement," as it is called—that is, the means by which these bones of ours, which form our arms and legs, can be moved and made useful. What makes the difference between ourselves and wooden dolls? Simply that we have muscles, or bundles of very minute fibres, which have the power of becoming longer or shorter at will. These muscles form the whole of what is usually known as "flesh." When you eat your meat at dinner you are eating certain muscles of an ox or sheep, or whatever the animal may be ; and an anatomist could give you the name of the particular muscle you might happen to be eating, and could tell you what bones it moved. It is hard to believe that the hard muscle which you can feel if you squeeze the upper part of the arm of a person who is in the habit of using his arms much is really the same as the meat which you can eat. If the animal whose flesh you are eating had been allowed to grow old, and, instead of being carefully fed and kept in a field where its food was close at hand, it had had to get food when it could, and, perhaps, walk many miles in the day, its flesh or muscle would be as hard as that of the arm you felt ; and if the owner of the arm had led an easy-going, luxurious life, and never done any work, that arm would have been as soft and tender as the muscle of the stall-fed ox. Some doctors say they can learn a great deal as to the state of a person's health by feeling their arm and noting whether the muscle feels soft and flabby, or firm and solid ; also walking behind people in the street this same doctor says he noted whether people's cheeks shook as they walked, and he always found in his experience that flabby muscles meant improper health, not that they were really ill, but that their health was not as good as it should be. Muscles are for the most part spindle-shaped—that is, narrower at either end, and thicker in the middle ; and when they contract to bend a bone they become shorter and thicker. You must bear in mind that the will is exercised over the muscle, and by making that shorter or longer it bends or straightens any two bones united by a joint, and therefore the power of the will over the bone is only indirect ; but you will have to know something about the nerves before you can properly understand this. Where the muscles become narrow, or pass over joints, they become harder, flatter, and broader, and are then called "tendons," or what you would call "leaders," and the reason why the muscle changed like this is that it may have a firmer hold, and not be torn off in violent exertion, and the tendons are *very* strong. I don't think I am exaggerating at all when I say they are the strongest tissues in the body. Even in cases of great violence it is very rare indeed to find these tendons actually torn, though they can be strained, and very painful it is when they are thus strained, far worse than when the bone is broken. If you hurt your wrist badly and strained these "leaders" you could feel them standing up like knots or hard cords, front and back, and they can be traced from the hand up the forearm, and find it connected with a muscle, and then, if you trace it downwards again, you will find it goes to the bones of the fingers. So remember that all through the body the tendons join the bones and the muscles. In every muscle you will find one end of it is more or less fixed, called the "origin," and the other end is joined on to a bone that moves, and therefore that end of the muscle is capable of more movement, and is called the "insertion." Take the arm, for instance—the muscle which is to move the forearm and allow you to bend it at will is fastened securely by its tendon to the shoulder ; that end is called the origin. The other end is fastened to the bone below the elbow, and is called the insertion. When you want to bend your arm you can feel no movement at all at the shoulder, but a great deal at the elbow. As the muscles are intended for motion, if they are not exercised they become weak and soft, and actually smaller, as you may see in a person who has been bed-ridden for a long time. If you feel that person's calves of the leg they will feel quite flabby. The muscle has wasted away, fat gets deposited amongst the fibres, its power becomes weaker, and any sudden strain on it might make it snap. A man who had not used his arm for throwing for many years once tried to throw a stone with force, so as to send it a long way, and his arm fell down helpless by his side. He had snapped the muscle, which had become weak from want of use, and now it did its work no more. Again, a muscle can be over-exercised, and then it becomes extremely hard, and tough, and red ; but when the violent exercise is removed the muscle will probably become as it was before, and will afterwards diminish in size.

### More Voyages in Vacation.—I.

Off Ushant, *February 28.*

Once again on board the dear old "Victoria," which is rapidly becoming a second home, so familiar is the trig, all spick-and-span yacht after our many experiences of her comforts, and her seaworthiness, as well as of the kindness and courtesy of her officers and crew. This time we are fleeing from the dreary, mirky, February weather—its glooms and its fogs—away to the blue Mediterranean ; turning our backs on all the ills belonging to a typical English spring. Not that our beginning is encouraging, for the cold at the present moment, when we find ourselves somewhere off the Isle of Wight, but not in sight of Ushant, is tremendous, and we are powdered over with fine, dry snow, in addition to which we are roughly knocked about by whirligigs of squally gusts. The air, however, is so bracing, so exhilarating, that it helps one to fight the cold, and, under mountains of wraps, we move about on deck greedy to get what sunshine there is to be had. Another reason is, to furtively take stock of our fellow-passengers—an entirely new set on this occasion—for, perhaps, nowhere are we mortals more thrown upon the tempers and idiosyncrasies of our fellows than 'board ship. As usual, we set off with the generally erroneous determination that there never was a more uninteresting collection gathered together, forgetting that the mere fact of each of us being on the defensive, so to speak, against each other must necessarily render us all at our worst. Time, however, will remedy all that, and the sooner the better, for the hours of a six-weeks' cruise will fly apace. Already, we can see that some friends of the second officer promise to be nice—the gentleman who wears a great fur coat and an eyeglass in one eye, and his wife, who wears a long fur coat and an eyeglass in the other. Their boy, who does not wear an eyeglass at all, cannot play bags, and, as we have set up some bean bags, he has got to be educated. Most of the ladies are *hors de combat* from sea-sickness, others are too cold to move out of their cabins, so we cannot, as yet, judge how they may turn out.

As we get further from England the weather becomes clearer and warmer. The snow ceases, the decks are dry, by the afternoon, and the ship looks more like our sprightly, little "Victoria," her pretty sails all set. It takes a little time to shake ourselves into our places; last night there was some grumbling regarding the dinner, and people appealed to me, as an old passenger, to know if the *ménage*

would improve. I was able to assure them it would, for did I not know the reason of the cold soup, etc.? Both cooks, I had been privately told, were what is called "gloriously on," a situation belonging to the unsettled state of a first evening—and never repeated. Twice have we tried to begin letter writing, but until a certain amount of rushing about had warmed our hands, they were quite unequal to holding a pen—from sheer cold. I could keep myself warm by incessantly walking about, but it would be fatiguing to walk *all* the way to the Mediterranean.

We shall be in the Bay to-night; the captain has been praying all week for an east wind, and we have one, the sea being quite calm. People are beginning to thaw; everyone is enquiring after everyone else.

"Hope your wife is not suffering from sea-sickness?" asks one gentleman of another. "No, thank you; she's suffering from a huge portmanteau which prevented her coming up all morning, she has been so busy unpacking it."

There are on board many of the types one sees in *Punch*—Sir Gorgius Midas among the rest—and a man whom Wendell Holmes would, of a certainty, call "the breast-pin"; he wears such a cats-eye and diamonds that "in its bright radiance and collateral light" he is personally altogether extinguished. Dinner time approaches, and I must walk a mile or two before it to get up an appetite. This evening I am going to change my place to another table, it being a sheer impossibility to eat forty-two dinners facing Sir Gorgius Midas, a fat German, and a breast-pin. Quite a little scene goes on about this settling of places. The captain asked one difficult-to-please youth where he *would* sit, and got for a reply that he didn't care where it was so long as it was near the present writer! Why *will* people make me so conspicuous? Truly, acquaintance makes advances by leaps and bounds 'board ship, for I and this youth never saw each other until yesterday, and already I have been asked to take out—to be married to him—the girl of his choice, if I go to Calcutta in October, as I half purpose. Indeed, I have been invited to go to Australia next Christmas, taking India on the way. How we talk, nowadays! Think of the uplifted-in-horror mittened hands of our great grandmothers could they hear us! Such a cruise, though, would be charming should it come to pass; but its cost would certainly take a big slice out of one's income—from £300 to £600, everybody declares. But, on the other hand, think, just think, what pleasure one would get for that sum!

At last, the question of places has been settled for the voyage, and I am especially content, having nice folk to right of me, nice folk to left of me. The saloon has been so stuffy on account of the people sitting in it the whole of the day that I have surreptitiously opened the top of a window. There *will* be a fuss if anyone find it out! I have taken care to sit in the draught myself, however, to prevent others doing so. I prefer even whooping-cough to asphyxia; that reminds me, I am popularly supposed to have had whooping-cough lately. However, it is gone, whatever it was; by any other name it would sound as bad, and hurt as little! Doctor M——, a nice old man, with whom I have made friends, has not had many patients as yet; one sea-sick lady has sent to ask him for anti-pyrin, but none of the crew seemed to have taken headers downstairs as they did on a former voyage for the benefit of another doctor we wot of. We are going through the bay right merrily. The ship rolls (it would be rather odd if she did not) here, but we are having an excellent passage, and shall be out of danger in a few more hours. Things are rather quiet among the passengers, who are "strangers yet." We have been playing bags a little, but without much enthusiasm. Never mind, that will come. One lady has hinted that she is dreadfully disappointed in her fellow-passengers, so, like Lord Dundreary's little bird, she "flocks all by herself in a corner!" There are only nine ladies to thirty-eight men, and five of the former have as yet been too ill to appear. Such a disparity makes us very precious, and it is to be hoped will not be too much for our light heads. We must try to bear our honours meekly.

It was three degrees warmer to-day at twelve o'clock than yesterday; the wind was beautifully clear, and not too cold for sunshine, so we have been able to sit out a great part of the day. Of course, we cannot expect halcyon weather at this time of year, but how I long for the warmth promised us. Time has gone frantic; my watch is already three-quarters of an hour fast; it was set at Fenchurch-street on starting, and its owner weighed (nine stone three pounds) in order to judge on return the result of the "Victoria's" ocean-cure. I have invested in a "Murray's Guide to the Mediterranean," which looks superlatively interesting, but all the same I mean to think for myself during this cruise about places and people both, and not be led about, like a blind man, by a string—that is, a guidebook. And there is no manner of doubt that when we Victorians get to find out which is who and who is which among us, we shall have a pleasant time. Meanwhile, the sea air, the best of remedies for sleeplessness, has made us all so drowsy that of all places it strikes us our berths are the most desirable. Therefore, good-night! And I promise more entertaining matter in my next, when we hope to have something to look at besides sea and sky.

### Everybody's Opinion.

*Correspondence on all subjects is invited, but we cannot in any way be responsible for the opinions expressed by our correspondents. No communications can be entertained if the name and address of the correspondent is not given, or unless one side of the paper only be written on.*]

### THE PENSION FUND.

Nurse M. S. writes: Being one of the first thousand, I cannot let the opportunity pass without expressing my thanks to those gentlemen who have so generously given to the National Pension Fund, and also to all who have given their valuable time for the benefit of nurses, whose minds are kept free from all anxiety during their professional duties in providing for sickness and old age.

### OUTDOOR UNIFORM.

Hypatia writes: May I submit to your readers the following questions, in the hope that their answers may decide a disputed point of professional etiquette: Several nurses in a hospital, where only indoor dress is given, are anxious to adopt an outdoor uniform, can they copy that worn by the lady superintendent and nurses of a small hospital close by? Have the latter a right to object, or is hospital uniform public property? [There is nothing absolutely wrong in copying the dress of another institution, but, as a matter of convenience and courtesy, it is better not to imitate that of a neighbouring hospital. It would be better to choose a bonnet and cloak of a different colour or style. Ed. Mirror.]

### ASYLUM ATTENDANTS.

An Asylum Attendant writes: A short time since, a letter from an asylum attendant appeared in your valued journal. Only those having experience as asylum nurses can fully understand the grievances we so justly complain of. Your correspondent says there is no training, and if your commissioners make enquiries her statement will be repeated by many nurses. I am an old asylum nurse, and I assure you I have never received any instructions, directly or indirectly, from a head attendant or doctor. We have to work in the dark, and guess at our duties till time teaches us. We have no lectures on insanity, and consequently the insane are not treated as they should be. A lunatic can attack a nurse if she has not strength to hold her own, and the chance is an attendant may be killed; many are injured. Dangerous patients should be put under restraint to preserve themselves, as well as to save a nurse from useless physical exertion and exhausting worry. Let all attendants be trained in asylums as nurse-attendants, and when qualified receive a certificate. Patient and nurse will then find life less painful.

### EXAMINATION PAPERS.

Nurse Rose writes: I am so delighted to see that my letter, which you so kindly published, really has made a beginning, and that many other nurses have answered your examination questions; I hope some of them date their answers from country workhouses. You would not wonder at my pleasure in the idea of a nursing correspondence being started if you lived, as I do, with nurses who never read anything but the worst kind of penny fiction. But I feel guilty as regards the bad habit of using a — instead of ; or : —life is so short! But I will reform before I send you another paper.

Sunshine writes: I like "A Country Nurse's" suggestion about the examination questions very much, and wish you would get up a competition for the best answers. I think it would be a great help to nurses. I like the "Word Competition," but the questions would be instructive as well as amusing. If you could arrange a competition, and would advise us what works to study, it would be a great kindness, and I believe you would have no lack of competitors.

### Appointments.

[It is requested that successful candidates will send a copy of their applications and testimonials, with date of election, to The Editor, The Lodge, Porchester-square, W.]

Adelaide Hospital, South Australia.—The committee in London, consisting of Sir Arthur Blyth, K.C.M.G., C.B., Mr. Timothy Holmes, Dr. Norman Moore, and Mr. Burdett, having selected three out of some hundreds of candidates for the post of medical superintendent, the board of management at Adelaide have given the appointment to Dr. R. H. Perks, M.D., F.R.C.S. Dr. Perks has had a wide and varied experience, having been house physician and house surgeon at Guy's Hospital, and resident medical officer to the Royal Albert Hospital, Devonport. A recent inspection of the Royal Albert Hospital confirms the view we have long held of the efficiency of this institution, where Dr. Perks has maintained a high standard of medical and surgical administration, and where the nursing leaves little, if anything, to be desired. Dr. Perks is a great ornithologist, and his collection of birds and eggs is not only extensive, but unique in its completeness and value. We congratulate the Adelaide Hospital Committee on their selection, and wish Dr. Perks a genuine success in his new field of labour. The colonies have long shown commendable zeal in the promotion of the highest efficiency in their hospital arrangements, and the steps taken to secure the best available man for this appointment are as commendable as they are noteworthy.

Queen's Hospital, Birmingham.—Dr. G. F. Crooke, late pathologist to the General Hospital, Birmingham, has lately been appointed physician to out-patients at the Queen's Hospital. Dr. Crooke has made himself extremely popular with the students and others with whom his official duties have brought him into contact. He is a graduate of Edinburgh, where he obtained a gold medal for his thesis. He was formerly house physician to the Seamen's Hospital, and afterwards medical superintendent to the Leeds Union Infirmary. Some time ago Dr. Crooke, in the performance of his duties, contracted severe blood poisoning, from which, however, he has now perfectly recovered.

### Vacancies.

**The following vacancies are announced:**

**Matron.**

Jaffray Suburban Hospital, Birmingham, £50.

**Night Superintendent.**

Royal Portsmouth, Portsea, and Gosport Hospital.

**Nurses.**

Bath Home for Trained Nurses.

Bexley Cottage Hospital, £25.

Bradford Infectious Hospital (assistant), £23.

Brighton Fever Hospital, £28.

Brompton Hospital for Consumption (several), £25.

Chelsea Hospital for Women (several), £25.

Heart Hospital, Soho.

Lock Hospital, Harrow-road.

Morley's Convalescent Hospital.

National Hospital for Paralysed and Epileptic (two), £25.

North Devon Infirmary.

Nursing Institution, 96, 97, 98, Wimpole-street, London, W., Mr. Wilson has several vacancies for thoroughly trained nurses.

Patricroft Hospital, Manchester, £20.

Peterborough Infirmary.

Queen Victoria Nursing Institution, Wolverhampton (several), £25.

St. John's Nursing Institute, Southport (three).

Victoria Home for Nurses, Bournemouth (several), £25.

West Derby Union Infirmary, Liverpool, £25.

**District Nurses.**

North London Nursing Association (several).
Scarborough Nurses' Institute.
Weston-super-Mare Nurses Institute.
York Nurses' Institution.

### Notes and Queries.

To Correspondents.—1. Questions may be written on post-cards. 2. Advertisements in disguise are inadmissible. 3. In answering a query please quote the number. 4. If a private answer is desired, a stamped addressed envelope must be forwarded.

Notice.—We must really ask our correspondents to be more particular in enclosing name and address. Constantly we receive queries or letters with no name attached.

**Queries.**

(86) Can anyone give me a remedy for cramp? Also say if a course of massage would be likely to do good to a case of chronic rheumatism. Patient aged 77. *A. S. A.*

(87) *G. D.* asks: Could any of your readers give me information about examinations for obtaining certificates for dispensing suitable for a lady?

(88) Can anyone recommend a good book for monthly nurses, also one on the feeding and management of infants? *Nurse May.*

(89) 1. Will you recommend me a medical dictionary, reliable, but not very expensive? 2. What general hospital would take a probationer to train, aged 30 years? *Dilemma.*

(90) Can you give me the address of some good training infirmaries or hospitals—medical, surgical, and fever? *Mary.*

(91) *White Face* asks: If a girl is very healthy, is she not constitutionally strong? And if she has muscular strength, would she not be considered a strong and healthy girl?

(92) Will any of your readers kindly give me information (1) as to whether there are any institutes or hospitals where ladies are trained, and receive a small salary? (2) How long the actual training takes before a nurse is qualified? (3) How long does it take to obtain an obstetrical diploma? Any information will be gladly received by *One desirous of entering the Nursing Profession.*

**Answers.**

[In consequence of the absence of the Editor of this department several answers are unavoidably held over.]

(78) If "W. M." takes my advice, and I have had experience in the matter, she will try to obtain the post she requires by answering the advertisements in this paper, or placing an advertisement in The Hospital or *Morning Post*, ending with (no agencies). In this way "W. M." will be spared journeys and much disappointment. *Private Nurse.*

### OBEDIENCE.

The duty of the nurse is included under the three heads—her obedience to the physician, her loyalty to the patient, and her abnegation of self. If she is true in these respects she cannot well fail in the discharge of her functions. She is the agent, the professional machine, to interpret and carry out the instructions she receives. No theories, no preconceived ideas of her own may stand in the way of her execution of orders when received from the source of authority. She is not responsible for the result so long as she adheres to the course laid down. When she departs from this track, she assumes personally, it may be, the issue of life and death, and arrogates to herself a position and a responsibility for which she has had no adequate training, and which she is not entitled to assume. Zeal, interest, her own opinion—none of these is a fitting excuse for disobedience. There is but one straight line, there is but one truth—there is but one obedience, and that is entire, complete.—*Dr. E. L. Keyes.*

